# Methylmercury-Mediated Oxidative Stress and Activation of the Cellular Protective System

**DOI:** 10.3390/antiox9101004

**Published:** 2020-10-16

**Authors:** Masatake Fujimura, Fusako Usuki

**Affiliations:** 1 Department of Basic Medical Sciences, National Institute for Minamata Disease, Kumamoto 867-0008, Japan; fujimura@nimd.go.jp; 2 Division of Neuroimmunology, Joint Research Center for Human Retrovirus Infection, Kagoshima University, Kagoshima 890-8544, Japan

**Keywords:** methylmercury, oxidative stress, binding affinity, redox signaling, selenoenzyme, nonsense-mediated mRNA decay, posttranscriptional defect, thiol antioxidant capacity, Keap1/Nrf2 pathway

## Abstract

Methylmercury (MeHg) is a well-known neurotoxicant that causes severe intoxication in humans. In Japan, it is referred to as Minamata disease, which involves two characteristic clinical forms: fetal type and adult type depending on the exposed age. In addition to MeHg burden level, individual susceptibility to MeHg plays a role in the manifestation of MeHg toxicity. Research progress has pointed out the importance of oxidative stress in the pathogenesis of MeHg toxicity. MeHg has a high affinity for selenohydryl groups, sulfhydryl groups, and selenides. It has been clarified that such affinity characteristics cause the impairment of antioxidant enzymes and proteins, resulting in the disruption of antioxidant systems. Furthermore, MeHg-induced intracellular selenium deficiency due to the greater affinity of MeHg for selenohydryl groups and selenides leads to failure in the recoding of a UGA codon for selenocysteine and results in the degradation of antioxidant selenoenzyme mRNA by nonsense-mediated mRNA decay. The defect of antioxidant selenoenzyme replenishment exacerbates MeHg-mediated oxidative stress. On the other hand, it has also been revealed that MeHg can directly activate the antioxidant Keap1/Nrf2 signaling pathway. This review summarizes the incidence of MeHg-mediated oxidative stress from the viewpoint of the individual intracellular redox system interactions and the MeHg-mediated aforementioned intracellular events. In addition, the mechanisms of cellular stress pathways and neuronal cell death triggered by MeHg-mediated oxidative stress and direct interactions of MeHg with reactive residues of proteins are mentioned.

## 1. Introduction

Methylmercury (MeHg) is a well-established neurotoxicant that affects various cellular functions depending on the cellular context and developmental phase. Severe MeHg-intoxication episodes in humans have been recognized in a number of countries, including Japan (Minamata disease) [1], Iraq [2], and the USA [3]. Minamata disease was named for the first case of MeHg poisoning due to ingestion of seafood contaminated by MeHg discharged from a chemical plant. The disease involves two characteristic clinical forms: fetal type and adult type depending on the exposed age. Fetal-type Minamata disease, which is caused by exposure to MeHg in utero, shows cerebral palsy-like clinical features with delayed psychomotor development [4]. In contrast, adult-type Minamata disease, which is caused by MeHg intoxication in adulthood, shows Hunter-Russell syndrome-like features [5]. The main lesions are found in the central nervous and peripheral sensory nervous systems [6,7]. The patients show neurological signs associated with such pathological lesions. MeHg toxicity is an environmental concern for human health, especially in susceptible populations who frequently consume substantial amounts of fish or fish predators [8]. 

To date, many studies on the pathogenetic processes caused by MeHg exposure have been reported using various cells and animal models. The pathological changes in MeHg toxicity do not correspond directly to the accumulation of Hg in tissues. In a MeHg-exposed subacute rat model (20 ppm MeHg in drinking water every day for 28 days), for example, the cerebellum showed the most severe pathology with a lower Hg concentration than that in the liver or kidneys, which displayed fewer pathological changes from high Hg content [9]. These findings suggest that cellular responsive conditions play a role in the development of pathogenetic changes due to MeHg exposure. Research progress has noted the importance of oxidative stress in the pathogenesis of MeHg toxicity. The early incidence of reactive oxygen species (ROS) has been reported in many MeHg intoxication experiments [10,11,12,13]. The elimination of ROS by co-treatment with radical scavengers can prevent MeHg-cytotoxicity in vitro and in vivo [9,11,14], but failure to protect cells against such early oxidative stress leads to further progression of MeHg-toxicity, such as the occurrence of subsequent endoplasmic reticulum (ER) stress and apoptosis [12].

It has been revealed that high affinity for selenohydryl groups, sulfhydryl groups, and selenides [15] plays a critical role in the incidence of MeHg toxicity [16,17,18]. Many antioxidant enzymes and proteins have thiol and selenol residues. The high affinity of MeHg for selenohydryl groups, sulfhydryl groups, and selenides results in the impairment of antioxidant enzymes and proteins as well as the subsequent disruption of antioxidant systems, which leads to MeHg-mediated oxidative stress. On the other hand, it has been clarified that MeHg can also directly activate an antioxidant-signaling pathway. The high affinity of MeHg for sulfhydryl groups can activate the cellular antioxidant transcription factor Nrf2 through the interaction of MeHg and Nrf2 regulator Keap1 [19]. Nrf2 activation and translocation to the nucleus induce downstream antioxidant proteins and enzymes [20,21,22].

Furthermore, MeHg has been shown to induce posttranscriptional defects in antioxidant selenoenzymes [23]. The high affinity of MeHg for selenohydryl groups and selenides causes intracellular relative active selenium (Se) deficiency. Such MeHg-induced Se deficiency leads to failure in the recoding of a UGA codon for selenocysteine and results in the degradation of antioxidant selenoenzyme mRNA by nonsense-mediated mRNA decay (NMD). NMD is a cellular mechanism that detects the premature termination codon (PTC) located 5′-upstream of the last exon-exon junction and degrades PTC-containing mRNAs [24]. Generally, PTC is recognized when it is located upstream of an exon–exon junction with a distance of at least 55 nucleotides [25]. Targets for NMD can include mutationally-induced nonsense or frameshift codons, upstream open reading frames, alternatively spliced or mis-spliced mRNA [26]. NMD has been considered an mRNA quality surveillance mechanism to protect an organism against deleterious dominant-negative or gain-of-function effects of truncated proteins that arise from PTCs.

The incidence of MeHg-mediated oxidative stress should depend on the individual capacities of the intracellular redox systems to respond to the results of the interactions among the aforementioned MeHg-induced events. Once MeHg-mediated oxidative stress occurs, it may trigger activation of various cellular signaling pathways leading to cellular damage. Recent works clarified the molecular mechanism of MeHg-induced apoptosis and cortical neuronal cell death caused by MeHg-mediated oxidative stress.

## 2. Disruption of the Cellular Redox Systems by MeHg Exposure

The critical role of oxidative stress in the pathogenesis of MeHg toxicity has been demonstrated both in vitro [11,12,27,28,29,30] and in vivo [9,31,32]. Many studies have reported an increase in ROS after MeHg exposure [10,13], and suppression of such increases by co-treatment with the antioxidant Trolox, sodium selenite, and ebselen have also been reported [12,23]. Oxidative stress is a condition in which the normal function of the redox network is disrupted. MeHg can disrupt many antioxidant proteins and enzymes in the cellular redox system because of the high affinity of MeHg for selenohydryl groups, sulfhydryl groups, and selenides [15,33]. In particular, the high affinity of MeHg for selenohydryl groups and selenides leads to intracellular relative Se-deficient conditions, resulting in enhanced degradation of antioxidant selenoenzyme mRNA targeted by NMD [23]. Defects in antioxidant selenoenzyme replenishment due to mRNA degradation should exacerbate MeHg-induced oxidative stress.

### 2.1. Suppression of Antioxidant Protein and Enzyme Activity by MeHg Exposure

Glutathione (GSH) and thioredoxin (Trx) systems, which are the central regulators of cellular redox status, include antioxidant proteins and enzymes with the selenohydryl group or sulfhydryl group at the redox-active centers. 

GSH is the most abundant thiol compound and the major antioxidant that functions as a redox buffer. MeHg interacts with GSH and forms a GSH–MeHg complex, which is excreted from the ATP-binding cassette sub-family C member 4 (ABCC4) transporter. GSH consumption under MeHg exposure elicited GSH synthesis through the upregulation of glutamate–cysteine ligase (GCL), a rate-limiting enzyme for GSH synthesis [34,35]. It has been reported that GSH content shows no change in the brain, liver, kidneys, and muscles [9,35] or an increase in the brain [34] in rat or mice MeHg-exposed models, suggesting the replenishment of GSH occurs during the consumption of GSH under MeHg exposure. Glutathione peroxidase (GPx), the most abundant selenoenzyme, plays a role in preventing the production of ROS by reducing hydrogen peroxide (H_2_O_2_) and free fatty acid hydroperoxides. Exposure to MeHg decreases GPx1 activity [9,36,37] and GPx1 mRNA in vitro and in vivo [23]. It has been reported that overexpression of GPx1 prevents MeHg-induced neurotoxicity in cultured cerebellar granule cells, suggesting that GPx1 plays a critical role in MeHg-mediated disruption of the cellular redox systems [37]. 

In contrast, another selenoenzyme, thioredoxin reductase (TrxR) and its substrate Trx, are known to be involved in the regulation of a large network of redox reactions, including metallothionein, ribonucleotide reductase, and redox-factor-1 (REF-1) [38]. REF-1 is known to maintain the cysteine residues of transcription factors, such as nuclear factor kappa B (NF-κB), Nrf2, and p53, in the reduced form required for DNA binding. The active site of TrxR has a redox-active selenothiol/selenylsulfide [39] and is known to be sensitive to MeHg [40]. Since selenols have a lower p*K*_a_ than thiols and are fully ionized to selenolates under physiological conditions [39,41], selenols are more reactive toward Hg [40]. The decrease in TrxR1 activity caused by MeHg exposure has been shown in vitro and in vivo [23,40,42]. The reduction in activity of the cellular Trx system under MeHg exposure has also been demonstrated [40]. Such damage to activity in the Trx system should lead to the disruption of a large network of redox reactions [38]. It is generally known that males are more vulnerable to MeHg toxicity than females. The recent study on litters from dam mice exposed to MeHg (5 ppm MeHg in drinking water from early gestational period until postnatal day 21) showed that basal levels of GPx1 and TrxR1 mRNAs in cerebrum were lower in males than in females and that the activities of TrxR, GPx1, and Gpx1 mRNA after MeHg exposure decreased at a greater rate in males than that in females [43]. The results suggest that sex differences in the antioxidant system may affect sex differences in the susceptibility to MeHg.

Manganese-superoxide dismutase (Mn-SOD) localized in mitochondria functions in preventing the production of ROS by reducing superoxide radicals. The study of the effect of MeHg on Mn-SOD in mouse brains revealed that MeHg reduced Mn-SOD activity whereas Mn-SOD mRNA levels and protein synthesis were not affected by MeHg administration [44]. It has been reported that MeHg interacts with Mn-SOD through a reactive sulfhydryl group [19]. A decrease in Mn-SOD activity caused by MeHg contributes to an increase in ROS. It has been reported that overexpression of Mn-SOD prevents MeHg toxicity in HeLa cells [45].

### 2.2. Basal Level of Antioxidant Enzymes Associated with MeHg Toxicity 

Fragile cells exposed to oxidative stress should be more damaged by MeHg. The main target organ of MeHg toxicity is the central nervous system. Adult-type Minamata disease caused the site-specific brain lesions in the cerebellum and cerebrum. Neurons in the cerebellar cortex are arranged in three layers. The innermost layer is densely packed with numerous, small neurons called granule cells (cerebellar granule cells). The middle layer contains Purkinje cells, and the outmost layer, or the molecular layer, contains stellate and basket cells. Among these cerebellar neurons, cerebellar granule cells are the most vulnerable to MeHg. Furthermore, autopsy studies of human cerebrum revealed that the lesions are localized in the deeper layers cerebrocortical neurons, especially layer IV, compared to the shallow layers cerebrocortical neurons [46,47]. Such site-specific cerebral lesions were also observed in MeHg intoxicated animal models of rats and mice [48,49].

We previously demonstrated that cerebellar granule cells susceptible to MeHg have lower in situ expression of Mn-SOD, GPx1, and TRxR1 mRNAs than cerebellar molecular layers and Purkinje cells, which are known to be resistant to MeHg [49]. Furthermore, our in situ analyses of antioxidative enzymes expression using quantitative reverse transcription polymerase chain reaction from laser micro-dissected mouse cerebral cortex samples and immunohistochemistry revealed that lower basal expression levels of Mn-SOD and GPx1 mRNAs and proteins in the cerebrocortical neurons of deeper layers than those in shallow layers. In addition, an increase in Mn-SOD mRNA expression induced by MeHg exposure is lower in deeper layers than that in shallow layers [50]. These findings suggest that the different antioxidative systems in situ, including basal levels of antioxidant enzymes, play a role in the site-specific neurotoxicity of MeHg in the brain.

### 2.3. Posttranscriptional Defects of Selenoenzymes

MeHg-mediated increases in intracellular ROS cause changes in antioxidant gene expression. Our previous study demonstrated that MeHg exposure upregulated Mn-SOD, copper, zinc (Cu, Zn)-SOD, catalase, and TrxR1 mRNAs [23]. The upregulation of these mRNAs was mediated by ROS because treatment with the antioxidant Trolox suppressed the increase in these mRNAs. In contrast, selenoenzyme GPx1 mRNA was downregulated despite its decreased activity in vitro and in vivo [23]. In addition, Trolox failed to rescue such GPx1 mRNA decrease. Our in situ antioxidative enzymes expression analyses using laser micro-dissected mouse cerebrocortical neuron samples also revealed downregulation of GPx1 mRNA [50]. This is intriguing because oxidative stress due to the general burden of H_2_O_2_ caused upregulation of GPx1 mRNA, indicating that the MeHg-induced GPx1 mRNA decrease is specific to the burden of MeHg [23].

GPx1 has a single selenocysteine (Sec), in which Se is co-translationally inserted. Sec is encoded by a UGA codon, which shares a common codon to function as a terminator for protein synthesis. The biosynthesis of Sec occurs on its tRNA (Sec tRNA ^[Ser]Sec^), unlike the other 20 amino acids. Once activated Se is donated to the structure, Sec is completed [51]. The insertion of Sec into protein requires the Sec insertion sequence (SECIS) [52], SECIS binding protein (SBP2) [53], and Sec-specific elongation factor [54,55]. Under Se deficiency, however, the UGA codon for Sec may be recognized as a nonsense codon, known as a PTC, due to the incomplete biosynthesis of Sec. mRNAs harboring PTCs are known to be deleted by NMD, an mRNA quality control mechanism that is executed when PTC is located sufficiently upstream of the exon–exon junction [24,56,57,58]. A previous report demonstrated that Sec on GPx1 mRNA that resides 105 nucleotides upstream of the sole exon–exon junction was recognized as a PTC and degraded by NMD under active Se-deficient conditions [59]. 

**Figure 1:**
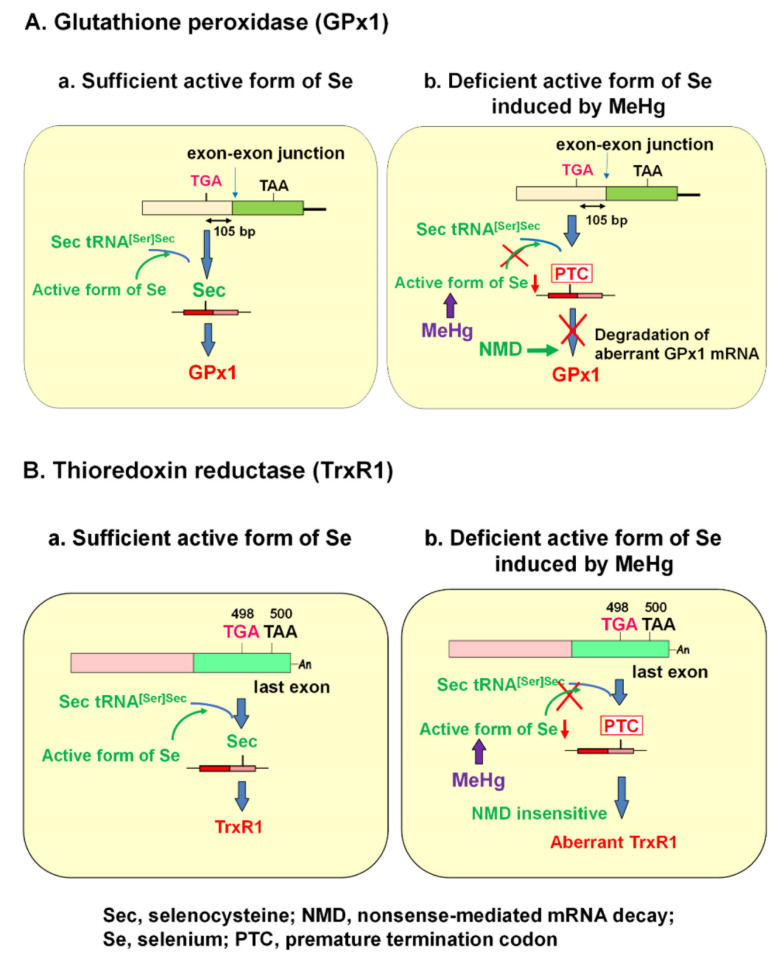
Posttranscriptional effect of methylmercury (MeHg) on antioxidant selenoenzymes. (**A**) Glutathione peroxidase 1 (GPx1). The encoded UGA codon for selenocysteine (Sec) resides 105 nucleotides upstream of the sole exon–exon junction. When a UGA codon is recognized as a Sec codon under sufficient active form of selenium (Se) (left panel), GPx1 is synthesized. However, since UGA codon is recognized as a nonsense codon under MeHg-induced active Se deficiency (right), GPx1 mRNA should be a natural substrate for nonsense-mediated mRNA decay (NMD; right panel). (**B**) Thioredoxin reductase 1 (TrxR1). The Sec codon UGA-498 resides in the last exon on TrxR1 mRNA; thus, TrxR1 mRNA cannot be a substrate for NMD even when a UGA codon is recognized as a nonsense codon under MeHg-induced Se deficiency and aberrant Trx1 is synthesized (right panel).

It is known that the selenohydryl group has a high affinity for Hg compared to those of the sulfhydryl and amino groups. The order of binding affinity of the coordination groups toward MeHg is as follows: SeH > SH ≥ Se-Se > NH_2_ > S-S [15]. The high affinity of MeHg for the selenohydryl group and selenide should cause relative intracellular Se-deficient conditions under MeHg exposure. Our previous study demonstrated that the MeHg-induced decrease in GPx1 mRNA is a post-transcriptional event by NMD, enhanced degradation of mRNA that is most likely mediated by cellular Se deficiency [23]. This finding was confirmed by two studies: (1) MeHg-induced decrease in GPx1 mRNA was rescued by pretreatment with sodium selenite, and (2) MeHg-induced decrease in GPx1 mRNA was inhibited in siRNA-mediated NMD component knockdown cells. In contrast to GPx1, mRNA of another antioxidant selenoenzyme, TrxR1, was not downregulated by MeHg exposure. The Sec codon UGA-498 on TrxR1 that resides in the last exon cannot be a substrate for NMD because at least one downstream intron is required to trigger NMD [25,60,61]. In theory, the TrxR1 protein synthesized by NMD-skipped TrxR1 mRNA should be truncated because the Sec codon is recognized as a nonsense codon under Se-deficient conditions. The different pathways involved in the synthesis of GPx1 and TrxR1 under the sufficient or MeHg-induced deficient active form of Se are summarized in Figure 1.

There are 25 known Se-containing proteins in mammals [62]. Under Se deficiency, it has been noted that selenoprotein expression has a hierarchy regarding the level of individual selenoproteins and Se in different organs [51]. The different distributions of MeHg accumulation after MeHg exposure [9] should also cause MeHg-induced intracellular Se-deficient conditions to be responsive to Se status in various tissues. In addition, the sensitivity of selenoenzyme activity to Se deficiency and mRNA turnover may affect selenoprotein expression. Many eukaryotic selenoproteins serve as antioxidant and redox proteins. Among them, the greater sensitivity of GPx1 activity to Se deficiency is attributed to an increased turnover in mRNA [63], resulting in enhanced degradation by NMD. Defects in the synthesis of GPx1 protein should exacerbate MeHg-mediated oxidative stress.

## 3. MeHg-Induced Mitochondrial Damage

Mitochondria is the most important sites of ROS generation. Imbalance of cellular ROS elimination system and RNA leakage by the disruption of mitochondrial function contributes to the occurrence of intracellular oxidative stress. It has been reported that isolated rat mitochondria show higher oxygen consumption levels and ROS production rates in the cerebrum and the cerebellum, which are the main lesion in MeHg toxicity, than in the liver [64]. In addition, it is known that GPx and SOD activities are lower in cerebrum and cerebellum than those in liver [64]. These findings may explain the difference in the distribution of MeHg-induced pathological changes.

### 3.1. Inhibition of Electron Transport Chain

The mitochondrial electron transport chain (ETC), composed of four respiratory enzyme complexes (I–IV) on the inner membrane, has been recognized as one of the most important sites of ROS generation [65,66]. Under physiological conditions, electrons are transferred fluently through the chains via adequate levels of ETC activity, resulting in the release of a minimum of mitochondrial ROS. When the ETC is damaged, however, electrons flowing through the chain will be disrupted, leading to the elevation of mitochondrial ROS leakage. If MeHg directly attacks the respiratory enzyme complexes or MeHg-mediated oxidative stress damages them, the function of ETC may be disrupted to produce excess ROS. The elevation of mitochondrial ROS leakage should cause oxidative stress and cell damage under the insufficient ROS elimination system. In the previous study, we demonstrated that MeHg treatment (orally administered at 5 mg/kg/day for 12 days) decreased mitochondrial enzymes (cytochrome c oxidase (CCO, complex IV) and succinate dehydrogenase (SDH, complex II)) activities in mitochondria-rich soleus muscle of rats [67]. Co-treatment with antioxidant Trolox clearly prevented such decrease in CCO and SDH activities despite the retention of MeHg, suggesting that MeHg-mediated oxidative stress caused a decrease in CCO and SDH activities [9]. MeHg-induced increase in the generation of H_2_O_2_ and O_2_^−^ in the mitochondria has been shown using extracted mitochondria from MeHg-exposed rat cerebellum [64]. Furthermore, the subsequent study showed that the complex II activity decreased in the cerebellum mitochondria and released cytochrome c in MeHg-exposed rats [68].

### 3.2. Mitochondria-Dependent Apoptotic Pathway

Many studies have shown that MeHg induces apoptosis due to both mitochondria- and ER-generated processes. Mitochondrial apoptotic pathway is governed by members of B-cell lymphoma-2 (Bcl2) protein family, which regulates mitochondrial outer membrane permeabilization (MOMP) [69]. Once MOMP occurs, cytochrome c is released from the mitochondria to the cytosol. Cytosolic cytochrome c interacts with apoptotic protease activating factor 1 (Apaf-1) and procaspase-9 to form a complex called apoptosome, which facilitates caspase-9 activation [70]. Caspase-9 is one of the initiators that cleave and activate the effector caspase-3, a central component of the apoptotic response.

It has been reported that MeHg induces activation of caspase-9 and caspase-3, high levels of cytoplasm cytochrome C, and apoptosis in cultured cortical neurons. Pre-treatment with Trolox significantly inhibited such neuronal apoptosis as well as mitochondrial dysfunction, suggesting these events were caused by MeHg-mediated oxidative stress [71]. MeHg-induced activation of mitochondria-dependent apoptotic pathway was also shown in the mouse cerebrum [72] and in developing rat hippocampus [73]. MeHg induced an increase in executioner caspase-3 by both mitochondrial-dependent caspase-9 and mitochondrial-independent caspase-8 in developing rat hippocampus. Studies using cultured neuronal cells suggested that ROS-mediated activation of ERK1/2 and p38 pathways regulated mitochondria-dependent apoptotic pathways that were involved in MeHg-induced neurotoxicity [72]. 

## 4. Cellular Stress Pathways Triggered by MeHg-Mediated Oxidative Stress

Oxidative stress generates not only free radicals but also nonradical oxidants. Free radicals tend to be reactive, and their initiation leads to macromolecular damage. In contrast, nonradical oxidants (e.g., H_2_O_2_, peroxynitrite, lipid hydroperoxide, and disulfides) are generated more than free radicals during oxidative stress, resulting in the disruption of redox signaling and physiological regulation pathways. Two major thiol- and selenol-containing GSH and Trx systems regulate such redox pathways, including receptor signaling (e.g., estrogen and glucocorticoid receptors), transcriptional regulation (e.g., NF-κB, Nrf2, p53, and HIF-1α), and apoptosis [38]. 

### 4.1. Apoptosis Signaling Pathway

MeHg disturbs intracellular redox systems through direct attacks on the cysteine and/or selenocysteine residues of antioxidant enzymes and a post-transcriptional effect on the major antioxidant selenoenzymes GPx1 and TrxR1 [23,40]. GPx1 is the most abundant selenoprotein that plays a critical role in the reduction in cellular H_2_O_2_. In contrast, TrxR1 transfers electrons from NADPH to thioredoxin, which in turn reduces thioredoxin peroxidase and other redox proteins [74]. It is known that TrxR1 can reduce some substrates other than Trx, including selenite [75], lipid hydroperoxides [76], and H_2_O_2_ [77]. As such, MeHg disturbs the GSH and Trx cellular redox systems at the early stage of cytotoxicity, which is followed by cellular stress responses. 

Trx is known to be a direct physiological inhibitor of apoptosis signal-regulating kinase-1 (ASK1) through binding of the active site dithiol with the N-terminus of ASK1 [78]. ASK1 activates c-Jun N-terminal kinase (JNK) and p38 MAP kinase pathways and is required for stress-induced apoptosis [79]. Oxidation of the Trx active site results in the release and activation of ASK1 followed by ASK1-dependent apoptosis. It has been demonstrated that MeHg exposure activates ASK1, subsequently resulting in activation of the stress-activated protein kinase (SAPK)/JNK pathways and apoptosis [12,29].

### 4.2. ER Stress

The ER is a membrane-bound organelle specialized for folding and post-translational maturation of secretory and membrane proteins. The ER redox state is linked to ER protein-folding homeostasis. Disulfide bond formation in the ER lumen is highly sensitive to altered redox balance, where both reducing and oxidizing reagents disrupt protein folding and cause ER stress [80]. In a stressed ER, dysregulated disulfide bond formation and breakage may result in ROS accumulation and cause oxidative stress. As such, ER stress can cause mitochondrial dysfunction and increase mitochondrial ROS production [81]. 

In a previous study, we showed that failure to protect cells against MeHg-mediated early oxidative stress triggers the subsequent ER stress and apoptosis [12]. A subsequent study showed that pretreatment with Trolox significantly blocked MeHg-induced ER stress, unfolded protein response activation, and apoptosis in neuronal cells [71], confirming that MeHg-mediated oxidative stress causes ER stress and apoptosis. In contrast, it has been reported that MeHg directly causes ER stress through interaction with protein disulfide isomerase (PDI) [18]. PDI localizes in the ER and catalyzes all of the reactions involved in native disulfide bond formation in the ER [82]. The oxidoreductase activity of PDI is derived from thiol groups of active site cysteines [83]. Furthermore, PDI has been identified as a multi-domain protein related to the cytoplasmic thioredoxin [84,85]. It has been demonstrated that the dysfunction of PDI enzymatic activity by the oxidative modification of active cysteine by nitric oxide (NO) induced by treatment with N-Methyl-D-aspartate causes the accumulation of newly synthesized unfolded protein in the ER lumen, resulting ER stress [86]. Similar to NO, the MeHg-modified C-terminal catalytic domain in PDI was detected using matrix-assisted laser desorption/ionization-time of flight mass spectrometry (MALDI-TOF/MS) analysis, suggesting that PDI is a target protein for MeHg in the ER [18]. In addition, treatment with MeHg significantly attenuated the enzymatic activity of PDI. These findings suggest that MeHg can also cause ER stress through the direct disruption of PDI function.

## 5. Activation of the Keap1/Nrf2 Pathway by MeHg Exposure

As a cellular compensatory function against oxidative stress, it has been known that MeHg upregulates antioxidant gene expression of GCL [34,35] as well as Mn-SOD, Cu, Zn-SOD, catalase, and TrxR1 mRNAs [23]. GCL is a rate-limiting enzyme for GSH synthesis and is known to be regulated by the transcription factor Nrf2 [87]. Nrf2 is an essential factor for the protective antioxidant response and detoxification against various environmental toxicants through the antioxidant responsive element (ARE)-mediated induction of enzyme genes [88]. Under physiological conditions, Nrf2 localizes in the cytoplasm and is inactivated through binding to Keap1 to repress its translocation to the nucleus [88]. Keap1 has highly reactive cysteine residues, which are the preferential targets of electrophiles and ROS. Electrophilic agents can functionally liberate Nrf2 from repression by Keap1, allowing Nrf2 to move to the nucleus and potentiate the ARE-mediated induction of enzyme genes [88].

It has been demonstrated by MALDI-TOF/MS analysis that MeHg reacts with Keap1 cysteine residues to cause a structural alteration [19]. Since pretreatment with the antioxidant Trolox could not activate Nrf2 in MeHg-exposed cells, MeHg may activate Nrf2 through direct interaction with the cysteine residues of Keap1 rather than MeHg-mediated oxidative stress [19]. Thus, MeHg exposure led to Keap1/Nrf2 dissociation, Nrf2 translocation to the nucleus, and ARE-mediated induction of oxidative stress enzyme genes, such as GCL and heme oxygenase-1 (HO-1) [20,21,22]. The cytoprotective role of Nrf2/Keap1 system to MeHg toxicity was confirmed by studies using Nrf2-overexpressed SH-SY5Y cells, and Nrf2- or Keap1-deficient mouse hepatocytes [20]. On the other hand, recent findings have shown that MeHg activates Nrf2 through the Keap1-independent pathway. It is known that Src subfamily kinase Fyn phosphorylates Nrf2, leading to nuclear export and degradation of Nrf2 [89]. It has been demonstrated that MeHg downregulates Fyn through the phosphorylation of Akt and glycogen synthase kinase 3 beta (GSK-3β), leading to sustained Nrf2 activity [90].

As such, MeHg can activate the antioxidant signaling pathway through direct interaction with the cysteine residues of the Keap1 and/or Akt/GSK-3β/Fyn pathway.

## 6. Neuronal Hyperactivity and Cell Death Triggered by MeHg-Mediated Oxidative Stress

Recently, the mechanism of neuronal cell death caused by MeHg-mediated oxidative stress has been clarified. Autopsy studies of human cerebrum revealed that the lesions were localized in the cerebrocortical neurons of deeper layers, especially layer IV, compared to shallow layers [46,47]. The neocortex is formed of six cortical layers, which are numbered I to VI from the outermost to the innermost. Layer IV, the internal granular layer, contains different types of stellate and pyramidal cells. The site-specific cerebral lesions were also observed in MeHg-intoxicated animal models. We previously reported that neuronal damage caused by MeHg is localized in layer IV of the cerebral cortex in adult mice, especially within the somatosensory cortex [48]. We further demonstrated that site-specific upregulation of c-fos and brain-derived neurotrophic factor (BDNF) preceded neuronal degeneration in layer IV of the cerebral somatosensory cortex of MeHg-intoxicated mice [91]. Layer IV has been shown to be mainly composed of excitable cells [92], suggesting that the characteristic of neuronal excitability may be related to susceptibility to MeHg neurotoxicity and subsequent damage. 

c-fos and BDNF are known to be proper markers of neural activity. In particular, c-fos expression analysis is carried out extensively to identify the site-specific neural activity in the brain [93,94]. It has been reported that the induction of c-fos expression is observed in brain-derived tissue exposed to several pathological agents. Exposure of a mouse hippocampal cell line to the 25–35 peptide fragments of A beta caused a rapid and sustained increase in nuclear c-fos immunoreactivity with a decrease in viability [95]. c-fos expression was also observed in the neocortex and paleocortex in picrotoxin-treated rats [96]. The expressions of c-fos and c-jun were significantly up-regulated with apoptosis by T-2 toxin treatment in human neuroblastoma cells [97]. In addition, it has been demonstrated that MeHg exposure significantly induces the expression of c-fos protein in cortex and hippocampus of rats [98]. As such, since c-fos expression has been observed during neuronal degeneration such as neuronal cell loss, and astroglia and microglia accumulation, c-fos may play a role as a trigger of neuronal degeneration. On the other hand, BDNF expression bears on neural activity concerning physiological activity and social stress [99,100]. 

The molecular mechanism of the relation between MeHg-induced upregulation of c-fos and neuronal cell death was investigated using *all-trans-*retinoic acid (RA) differentiated SH-SY5Y cells, which show neuron-like morphological changes and express neuron/synapse markers for cerebrocortical neurons [101]. Time course studies revealed that MeHg-induced upregulation of c-fos preceded neuronal cell death in RA-differentiated SH-SY5Y cells. We demonstrated early expression of the oxidative stress marker thymidine glycerol followed by activation of p44/42 mitogen-activated protein kinase (MAPK) and p38 MAPK, and an increase in cAMP response element binding protein (CREB) pathways. The antioxidants Trolox and edaravone significantly suppressed such MeHg-induced thymidine glycerol expression, p38 MAPK-CREB pathway activation, and neurotoxicity. Furthermore, treatment with SB203580, a specific inhibitor of p38 MAPK, significantly blocked the upregulation of c-fos and neuronal cell death. These results suggest that MeHg-induced oxidative stress and subsequent activation of the p38 MAPK-CREB pathway contribute to cerebrocortical neuronal hyperactivity and subsequent site-specific neuronal cell death.

## 7. Biomarkers for Ongoing MeHg-Induced Oxidative Stress

The Hg level in the hair, blood, or nails is known to be a useful biomarker to assess the MeHg body burden for a period of several months prior to sample collection, and the fetal umbilical cord is useful to assess the MeHg burden during fetal development. However, neither of these biomarkers sufficiently reflects individual toxic effects because MeHg toxicity depends on individual susceptibility to MeHg in addition to the MeHg burden. An assessment of MeHg in preserved umbilical cords collected in the Minamata region of Japan demonstrated that some patients with adult-type Minamata disease or mental retardation had MeHg concentrations that were as high as fetal-type Minamata disease despite not showing delayed psychomotor development [4], which suggests that the individual protective capacity against the MeHg body burden plays an important role in the severity of MeHg toxicity.

As mentioned above, MeHg-induced oxidative stress is generated by disruption of the antioxidant system caused by the high affinity of MeHg for selenohydryl groups, sulfhydryl groups, or selenides. Such characteristics may allow plasma thiols and selenoproteins to be used as biomarkers for individual susceptibility to MeHg and thus predict the early effects of MeHg intoxication. Thiol-disulfide pools in plasma can be related to systemic oxidative stress through continuous interactions with tissues and organ systems [102]. Therefore, we assessed the plasma levels of three oxidative stress markers, dROMs (diacron reactive oxidant metabolites) as derivatives of reactive oxygen metabolites, -SHp (thiol antioxidant barrier) as an overall measure of thiol antioxidants, and BAP (biological antioxidant potential) as an overall measure of antioxidants. We also assessed plasma selenoproteins, glutathione peroxidase (GPx3), and selenoprotein P1 (SeP1). We used MeHg-intoxicated model rats (20 ppm MeHg in drinking water every day for 28 days) that showed neuropathological changes after 4 weeks of MeHg exposure and compared the results to those obtained from rats treated with lead (Pb) acetate or cadmium (Cd) chloride, which have been reported to cause oxidative stress in tissues [103,104,105]. Three plasma oxidative stress markers were measured using a free radical elective evaluator (FREE) (Diacron International srl, Grosseto, Italy) and dedicated reagents. In this measurement, dROM quantifies the metabolite ROOH to evaluate oxidative stress, -SHp was measured using Ellman’s reagent (5,5′-dithiobis-2-nitrobenzoic acid), and BAP quantifies the reducing power of endogenous antioxidants to iron, which acts as a sensitive antioxidant. Such measurements of systemic oxidative stress markers using FREE have already been used clinically [106,107]. In this study, we identified the decreased capacity of -SHp, GPx3 activity, and SeP1 as useful potential plasma biomarkers of ongoing MeHg cytotoxicity [108]. Among them, the -SHp level significantly decreased 2 weeks after MeHg exposure, which is an early stage at which no systemic oxidative stress, histopathological changes, or clinical signs were detected. These findings suggest that the -SHp level is useful for assessing the early effects of MeHg exposure and subsequent changes in structure/function induced by MeHg intoxication. On the other hand, exposure to Pb or Cd did not alter oxidative stress markers in the plasma, although glial fibrillary acidic protein immunolabeled astrocytes were detected in the cerebellum of Pb-treated rats as previously reported [109,110], and the doses of Cd used in the study were reported to induce oxidative stress or neurotoxicity in previous studies [111,112,113]. In contrast to MeHg, an increase in SeP1 was observed in both Pb- and Cd-treated rats. The disruption of the antioxidant system caused by the high affinity of MeHg for the selenohydryl and sulfhydryl groups is a specific feature of MeHg-induced oxidative stress. Plasma biomarkers reflecting such features are useful for the evaluation of ongoing MeHg-mediated oxidative stress.

## 8. Prevention against MeHg-Induced Cytotoxicity

Based on the findings of the mechanism of MeHg toxicity, there have been many reports on effective treatments to prevent cytotoxicity triggered by MeHg-induced oxidative stress in vitro and in vivo. MeHg toxicity can be prevented by co-treatment or pretreatment with below-mentioned chemicals. Effective treatments to prevent in vivo toxicity triggered by MeHg-induced oxidative stress are summarized in Table 1.

### 8.1. Radical Scavenging Chemicals

The early incidence of ROS leads to further progression of MeHg toxicity. Therefore, a number of radical-scavenging chemicals have been tried to prevent MeHg toxicity in vitro and in vivo. They include α-tocopherol [14,119], Trolox (a water-soluble derivative of vitamin E) [9,30], n-propyl gallate [30,120], and tocotrienol (unsaturated vitamin E) [119]. Inhibitory effect of α-tocopherol on MeHg-induced oxidative stress has been demonstrated in rats, which were treated with 5 mg/kg MeHg for 12 consecutive days and 150 mg/kg α-tocopherol for 20 consecutive days after initial MeHg administration. α-tocopherol-treated rats showed decreased lipid peroxidation and manifestation of clinical signs (hind limb crossing sign and ataxic gait) compared to rats with MeHg alone [14]. Co-treatment with Trolox protected MeHg-treated rat skeletal muscle (20 ppm MeHg in drinking water every day for 28 days) against the decrease in mitochondrial electron transport system enzyme activities (cytochrome c oxidase and succinate dehydrogenase) despite the retention of MeHg. In addition, Trolox was effective for protecting cerebellum from MeHg-induced apoptosis in rats [9]. Pre-treatment with n-propyl gallate protected cultured human cerebellar granular cells established from fetal brain tissue from MeHg cytotoxicity [120].

### 8.2. Replenishment of GSH, Se, or GPx1

GSH interacts with MeHg to form a GSH-MeHg complex which is excreted from the ABCC4 transporter. Further, GSH functions as a redox buffer, leading to GSH consumption under MeHg exposure. N-acetyl-L-cysteine (NAC), an amino acid derivative, provides cysteine for GSH production. In addition, it has been shown that NAC can scavenge oxidants directly, reducing hydroxyl radicals and hypochlorous acid [121]. Co-treatment with NAC protected cultured cells against MeHg-cytotoxicity [11,117]. It has been reported that co-treatment with NAC reduces MeHg-induced neurotoxicity in the developing rat hippocampus [117]. Furthermore, post-treatment with NAC accelerated urinary MeHg excretion in mice [122]. 

The high affinity of MeHg for the selenohydryl group and selenide should cause relative intracellular Se-deficient conditions, leading to the degradation of antioxidant selenoenzyme mRNA by NMD. Therefore, the replenishment of Se should improve such impairment. It has been reported that pretreatment with sodium selenite rescues MeHg-induced downregulation of GPx1 mRNA, an increase in intracellular ROS, and MeHg-induced decrease in TrxR1 activity in vitro [23]. Co-treatment with sodium selenite can suppress MeHg-mediated neurotoxicity in rats [114] and fetotoxicity in mice [115]. Co-treatment with selenomethionine, a food-based Se, prevented MeHg-induced neuronal degeneration and reactive astrocytosis in a postnatal rat model, suggesting that dietary Se is useful for the protection of neurons against MeHg cytotoxicity [116].

Probucol is a phenolic lipid-lowering drug with anti-inflammatory and antioxidant properties. Probucol has been shown to increase GPx1 activity in the rat heart and prevent adriamycin-induced myocardial toxicity [123]. Co-treatment with probucol protected mouse cerebellar granular cells against MeHg cytotoxicity, which was correlated with increased GPx1 activity and decreased lipid peroxidation [37]. 

### 8.3. Seleno-Organic Compound Ebselen

Ebselen, a seleno-organic compound, exhibits GPx1 mimic activity and can quench free radicals and singlet oxygen [124]. Furthermore, ebselen is an excellent direct substrate for mammalian TrxR and Trx [125,126]. Ebselen treatment does not increase the amount of bioavailable Se [124] but generates the selenol form of the compound [125]. A number of reports have demonstrated the effectiveness of ebselen. MeHg-induced glutamate release from rat brain synaptosomal preparations was inhibited by co-treatment with ebselen [127]. Co-treatment with ebselen could also protect MeHg-induced glutamate uptake inhibition in rat cerebral cortical slices [128]. Furthermore, pre-treatment with ebselen suppressed MeHg-induced inhibition of glutamine uptake in rat neonatal cortical astrocytes [129]. We also demonstrated that co-treatment with ebselen effectively suppressed the downregulation of GPx1 mRNA and the ROS increase, and finally cytotoxicity after MeHg exposure in vitro [23].

### 8.4. Nrf2 Activators

It has been reported that isothiocyanates causes release of Nrf2 from sequestration by Keap1, and its subsequent translocation into the nucleus, resulting in the ARE-mediated induction of oxidative stress enzyme genes [130]. ARE-mediated genes include GCL, glutathione S-transferase, and multidrug resistance-associated protein, which are associated with MeHg excretion. It has been reported that pretreatment with isothiocyanates 6-methylsulfinylhexyl isothiocyanate (6-HITC) or sulforaphane (SFN) before MeHg exposure suppresses Hg accumulation and cytotoxicity in mouse hepatocytes [118]. Furthermore, SFN treatment prior to administration of MeHg also suppressed Hg accumulation in the brains of mice and clinical sign hind-limb flaccidity [118].

### 8.5. ER Stress Preconditioning

Cells and tissues can be protected against potentially lethal stress by pre-exposure to the same or different milder stress. Preconditioning cytoprotection has been described in ischemic preconditioning against myocardial infarction [131,132] or delayed neuronal cell death [133] and ER stress preconditioning against renal epithelial cell oxidative injury [134] or cardiomyocyte oxidative injury [135]. We demonstrated that cells preconditioned with an inhibitor of ER Ca^2+^-ATPase, thapsigargin, showed resistance to MeHg-induced cytotoxicity through favorable stress responses, which included phosphorylation of eukaryotic initiation factor 2 alpha, accumulation of activating transcription factor 4, upregulation of stress-related proteins, and activation of the extracellular signal-regulated kinase pathway [136].

## 9. Conclusions

High affinity for selenohydryl groups, sulfhydryl groups, and selenides plays a critical role in the incidence of MeHg-mediated oxidative stress. It causes the impairment of many antioxidant enzymes and proteins, resulting in the disruption of antioxidant systems. On the other hand, MeHg can also activate an antioxidant signaling pathway. Cellular antioxidant transcription factor Nrf2 can be activated through the direct interaction of MeHg and Nrf2 regulator Keap1. Furthermore, MeHg-induced Se deficiency due to the high affinity of MeHg for selenohydryl groups and selenides leads to failure in the recoding of a UGA codon for Sec and results in the degradation of antioxidant selenoenzyme mRNA by NMD. The incidence of MeHg-mediated oxidative stress arises from the interactions of individual intracellular redox systems and the aforementioned MeHg-mediated events. MeHg-mediated oxidative stress causes apoptosis through activation of ASK1 and the subsequent SAPK/JNK pathways. MeHg induces site-specific cerebrocortical neuronal cell death, which was observed in layer IV of the cerebral cortex mainly composed of excitable cells. It has been demonstrated that the mechanism of MeHg-induced site specific cortical neuronal damage is caused by cerebrocortical neuronal hyperactivity triggered by MeHg-mediated oxidative stress and subsequent activation of the p38 MAPK-CREB pathway. 

**Figure 2:**
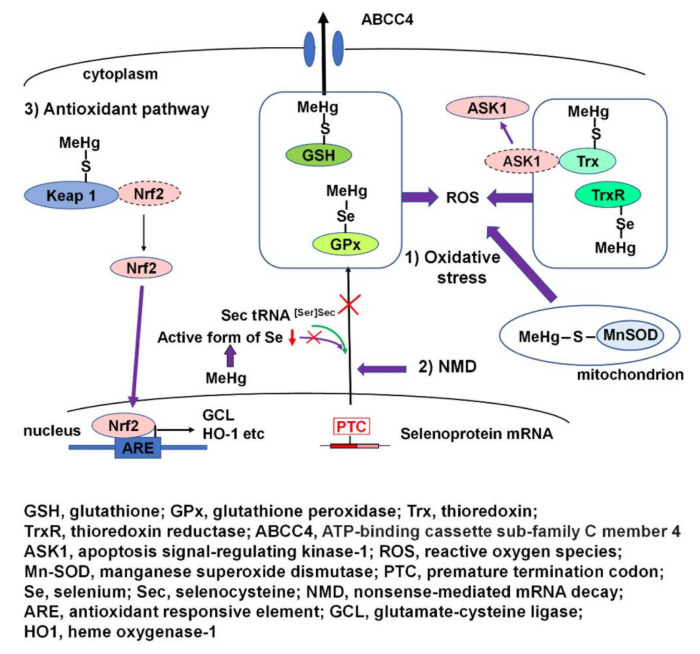
Schematic overview of MeHg-mediated oxidative stress and activation of the antioxidant Nrf2 pathway. (1) Thiols and selenols of many antioxidant proteins and enzymes of glutathione (GSH) and thioredoxin (Trx) cellular redox systems, such as GSH, GPx1, and TrxR, interact with MeHg, resulting in the disruption of the cellular redox system and the incidence of oxidative stress. The interaction of MeHg and Trx results in release and activation of apoptosis signal-regulating kinase-1 (ASK1) followed by ASK1-dependent apoptosis. In addition, interaction of mitochondrial Mn-SOD with MeHg may contribute to an increase in reactive oxygen species (ROS). (2) MeHg-induced relative intracellular Se deficiency causes failure in the recoding of a UGA codon for Sec because of active Se deficiency and results in the degradation of antioxidant selenoenzyme mRNA by nonsense-mediated mRNA decay (NMD). (3) The interaction of MeHg and Nrf2 regulator Keap1 leads to the activation of antioxidant transcription factor Nrf2, resulting in the antioxidant responsive element-mediated induction of oxidative stress enzyme genes, such as glutamate-cysteine ligase (GCL) and heme oxygenase-1 (HO-1).

A schematic overview of MeHg-mediated oxidative stress and activation of the antioxidant Nrf2 pathway is presented in Figure 2.

## Figures and Tables

**Table 1:** In vivo protection against methylmercury (MeHg) toxicity.

Treatment	Function	Animal	Effectiveness	Ref.
Trolox	radical scavenging	rat	cytotoxicity	[9]
			clinical feature	
α-tocopherol	radical scavenging	rat	cytotoxicity	[14]
			clinical feature	
sodium selenite	replenishment of selenium	rat	clinical feature	[114,115]
selenomethionine	replenishment of selenium	postnatal rat	clinical feature	[116]
NAC	replenishment of GSH	rat	DNA synthesis	[117]
			hippocampal cell number	
sulforaphane	Nrf2 activator	mice	cellular Hg content	[118]
			clinical feature	

NAC, N-acetyl-L-cysteine; GSH, glutathione.
